# Iron overload in a teenager with xerocytosis: the importance of nuclear magnetic resonance imaging

**DOI:** 10.1590/S1679-45082013000400022

**Published:** 2013

**Authors:** Reijâne Alves de Assis, Carolina Kassab, Fernanda Salles Seguro, Fernando Ferreira Costa, Paulo Augusto Achucarro Silveira, John Wood, Nelson Hamerschlak

**Affiliations:** 1 Hospital Israelita Albert Einstein, São Paulo, SP, Brazil; 2 Universidade Estadual de Campinas, Campinas, SP, Brazil; 3 University of Southern California, California, United States

**Keywords:** Anemia, Iron overload, Magnetic resonance imaging, Chelation therapy, Case reports

## Abstract

To report a case of iron overload secondary to xerocytosis, a rare disease in a teenager, diagnosed, by T2* magnetic resonance imaging. We report the case of a symptomatic patient with xerocytosis, a ferritin level of 350ng/mL and a significant cardiac iron overload. She was diagnosed by T2* magnetic resonance imaging and received chelation therapy Ektacytometric analysis confirmed the diagnosis of hereditary xerocytosis. Subsequent T2* magnetic resonance imaging demonstrated complete resolution of the iron overload in various organs, as a new echocardiography revealed a complete resolution of previous cardiac alterations. The patient remains in chelation therapy. Xerocytosis is a rare autosomal dominant genetic disorder characterized by dehydrated stomatocytosis. The patient may present with intense fatigue and iron overload. We suggest the regular use of T2* magnetic resonance imaging for the diagnosis and control of the response to iron chelation in xerocytosis, and we believe it can be used also in other hemolytic anemia requiring transfusions.

## RESUMO

Relatar um caso de sobrecarga de ferro secundária à xerocitose, uma doença rara, em uma adolescente, diagnosticada por meio de ressonância magnética em T2*. Relatamos o caso de uma paciente sintomática com xerocitose, nível de ferritina de 350ng/mL e sobrecarga de ferro cardíaca significativa. Ela foi diagnosticada por ressonância magnética em T2* e recebeu terapia de quelação. Análise por ectacitometria confirmou o diagnóstico de xerocitose hereditária. Na sequência, a ressonância magnética em T2* demonstrou resolução completa da sobrecarga de ferro em vários órgãos e novo ecocardiograma revelou resolução completa das alterações cardíacas anteriores. A paciente permanece em terapia de quelação. Xerocitose é uma desordem genética autossômica dominante rara, caracterizada por estomatocitose desidratada. O paciente pode apresentar fadiga intensa e sobrecarga de ferro. Sugerimos o uso regular de ressonância magnética em T2* para o diagnóstico e controle da resposta à quelação de ferro em xerocitose e acreditamos que o exame pode ser útil também em outras anemias hemolíticas que necessitam de transfusões.

## INTRODUCTION

The hereditary form of dehydrated stomatocytosis (xerocytosis) is an autosomal dominant genetic disorder characterized by increased permeability (leaks) of the erythrocyte membrane potassium pump, producing dehydration of red blood cells. The word “stomatocytosis”, initially described in 1974^(1,2)^, derives from the morphology of red cells in a peripheral blood smear, which resembles the mouth or lips. Other clinical situations may present this cell morphology, such as liver diseases or other hemolytic anemias, but these disorders present no change in the permeability of the erythrocyte membrane^(3)^.

Xerocytosis is a rare disorder, with an incidence of about 1 in 50,000 live births. This is 10 to 20 times lower than that of hereditary spherocytosis. The most frequent changes in xerocytosis were mapped as allele alterations of chromosome 16q23-qter^(3–5)^. It may be considered a pleiotropic syndrome in the sense that the gene defect affects more than the red cell^(6)^. There have been reported cases of xerocytosis with recurrent abortion and hydropsfetalis showing no response to the use of intrauterine transfusion, which is spontaneously resolved after birth^(1,6)^. Another frequent clinical complication is an increased tendency towards thromboembolic events in patients with xerocytosis after splenectomy. The pathophysiological mechanism of these events has not been well-defined, but studies show that the erythrocytes of these individuals are more adherent to the endothelium. The correct diagnosis of these membrane disorders can modify the clinical course because the diagnosis of spherocytosis leads to splenectomy, which is not indicated in xerocytosis^(1,3,5)^.

Laboratory analysis may reveal macrocytosis, elevated mean corpuscular hemoglobin concentration (MCHC), reticulocytosis, decreased osmotic fragility (increased resistance to osmotic lysis) and high levels of serum potassium in sequential measurements at regular intervals (pseudohyperkalemia)^(1,4)^. The cells show greater resistance to fragmentation when submitted to high temperatures (46 to 49°C) when compared with normal erythrocytes^(1)^. In contrast to the hyper-hydrated form of hereditary stomatocytosis, a xerocytosis peripheral blood cell smear shows spikes and fewer stomatocytes. The number of stomatocytes present in xerocytosis is usually less than 10% of red cells^(6)^. Ektacytometry and serial measurements of potassium in the same sample (showing pseudohyperkalemia) can confirm diagnosis^(4)^. Osmotic gradient ektacytometry shows a curve that is absolutely specific (the maximum deformability index is normal; the “hypo-osmotic point”, the “hyper-osmotic point”, osmotic fragility and cell hydration are decreased)^(5)^.

Clinically, xerocytosis is manifested by mild to moderate hemolytic anemia. The patient may present fatigue disproportional to the degree of anemia, which seems to be due to lower levels of 2,3-diphosphoglycerate (2,3-DPG) and elevated consumption of adenosine triphosphate (ATP) as a result of increased Na/K pump activity, which can affect oxygen affinity^(3)^. Affected individuals usually present mild to moderate anemia and do not require frequent transfusions. Even so, they tend to have a significant iron overload, and the mechanism for this has not yet been clarified^(4,5)^. There seems also to be a lack of correlation between levels of serum ferritin and other diagnostic results of iron overload, including T2* magnetic resonance imaging (MRI T2*) in cases with cardiac overload. This has been reported in studies of MRI T2* in patients with thalassemia major^(7,8)^.

## CASE REPORT

A 17-year-old Caucasian female patient was admitted to in March 1999, complaining of symptomatic anemia and presenting features of hemolysis, jaundice, and choluria, and having needed sporadic transfusions since childhood. She also reported a history of splenectomy and cholecystectomy at another treatment facility, as well as a liver biopsy (performed in March 1996), revealing hemosiderosis. She denied a similar family history. She had a history of neonatal ascites.

Laboratory results showed a hemoglobin concentration of 9.8g/dL, a mean corpuscular volume (MCV) of 113.2fl and a MCHC of 31.7g/dL. Hemoglobin electrophoresis and the glucose-6-phosphate dehydrogenase (G6PD) test were normal. The molecular analysis for alpha thalassemia showed no mutations, no unstable hemoglobin was detected, screening of paroxysmal nocturnal hemoglobinuria using the Ham test (lysis by acidified serum), screening of sucrose, and subsequent flow cytometry, were all negative. The bone marrow findings showed only hypercellularity in the erythroid series. The osmotic fragility curve suggested increased globular resistance. The transferrin saturation was 73% and the serum ferritin level was 350ng/mL. There were no mutations for hemochromatosis (C282Y and H63D) by polymerase chain reaction (PCR).

In April 2001, the patient had arrhythmia and showed signs of systolic heart failure, which was confirmed by echocardiography. She also showed signs of pulmonary hypertension. As the patient was symptomatic, treatment with aldactone (25mg/day), amiodarone (200mg/day) and digoxin (0.25mg/day, with control of serum in regular doses) was initiated with good response. Blood collected after the heart failure episode showed thrombocytosis, which was attributed to the previous splenectomy.

**Figure 1:**
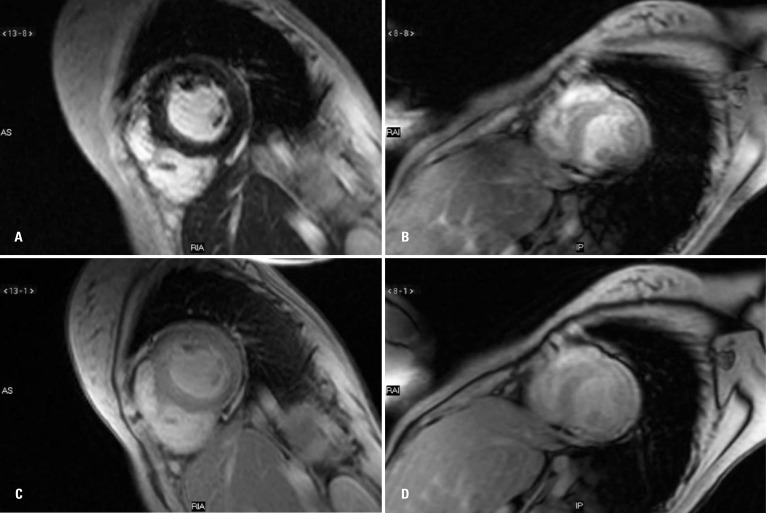
Magnetic resonance exam. (A and B) Pre-treatment image took during combination therapy of iron chelators (2005); fall of myocardial T2* sequence signal, T2*=6.5ms*; (C and D) Image after treatment, showing no significant drop of signal, which shows normal examination for evaluation of iron overload, T2*=45ms. Note that the value of T2* is inversely proportional to iron overload; the lower the value, the greater the load

In April 2005, nearly 4 years after the onset of the cardiac symptoms and specific treatment for heart insufficiency, MRI T2* was requested for the quantification of iron deposits in the heart, liver and pancreas. The indices were: heart, 6.5ms; liver, 6.7ms (4.46mg Fe/g liver); and pancreas, 9.1ms, with positive results for iron overload in the heart, liver (Figure 1) and pancreas and a serum ferritin level of 267ng/mL.

Chelation with deferiprone (3,500mg/day) was introduced orally in combination with subcutaneous deferoxamine (2.0g/day) for periods of 10 hours a day by infusion pump. The patient was intolerant to iron chelation, presenting urticarial, erythematous, and pruritic reactions at the site of deferoxamine application (local hypersensibility). The iron chelation therapy was temporarily suspended and was reintroduced only 1 month later with good tolerance, using deferoxamine (2.0g/day) alone by subcutaneous application on alternating days. The serum ferritin dosage during the resumption of deferoxamine use was 240ng/mL.

The patient remained without an etiologic diagnosis. The clinical picture of hemolytic anemia, hemochromatosis, and the morphological findings in peripheral blood (which revealed stomatocytes and acanthocytes) suggested the possibility of stomatocytosis. Ektacytometric analysis was performed, and with the possibility of xerocytosis in mind, serial measurements of the patient's and her parents' potassium were requested. Pseudohyperkalemia was found in the patient's sample, as shown in table 1, confirming the diagnosis of hereditary stomatocytosis, dehydrated form (xerocytosis).

**Table 1:** Analysis of serum potassium at 0, 2, 4 and 6 hours in the patient and parents samples, showing pseudohyperkalemia in the patient's samples

	Time			
	Initial	2 hours	4 hours	6 hours
Patient	4.6	5.7	5.9	6.3
Father	4.4	4.3	4.3	4.4
Mother	4.3	4.3	4.2	4.3

**Figure 2:**
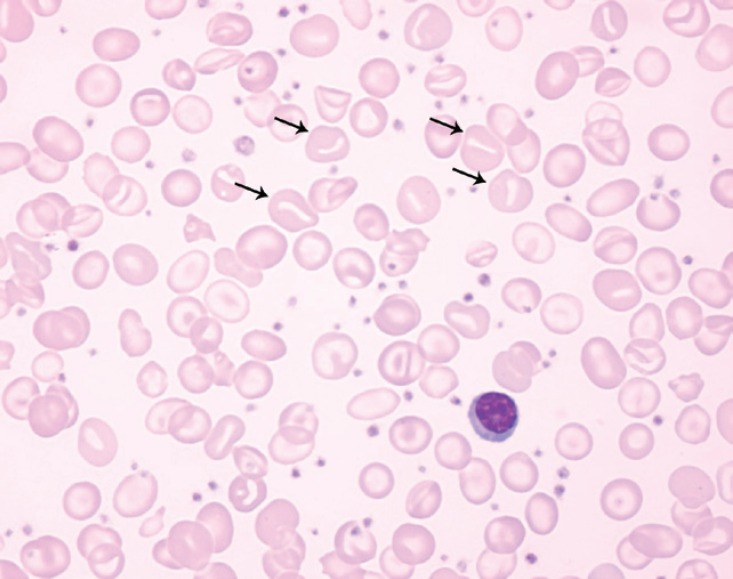
Cytology smear of peripheral blood of the patient with stomatocytes (arrows). Leishmandye, 100 x

Control with MRI T2* was completed in February 2006, about 8 months after chelation therapy, showing a reversal of the signs of liver overload (normalization of function) and improvements in pancreatic and cardiac functions (heart: 13.8ms; liver: 23.5ms; pancreas: 14ms, table 2). Iron chelation was continued with deferoxamine until April 2007, when it was supplemented and then replaced by deferiprone 25mg/kg orally, three times a day or 75mg/kg/day. Subsequent evaluation by MRI T2*, in April 2008, revealed a normalization of measures in all areas, as shown in figure 2.

**Table 2:** Complete recovery of cardiac iron overload, liver and pancreas as measured by MRI T2* and reflected in an improvement of cardiac function measured by echocardiography after chelation therapy

Measurement	Date				
	4/20/05	3/13/07	3/18/08	7/14/09	6/10/10
Left ventricular diastolic diameter (3.6–5.2cm)	5.2	4.8	5.2	4.8	5
Left ventricular systolic diameter (2.6–3.4cm)	3.6	2.9	2.9	3.2	3.1
Ejection fraction (>0.55%)	0.58	0.70	0.75	0.62	0.68
Left ventricular mass index (<96g/m^2^, female)	97	109	141	116	87
Aortic root (2.1–3.7cm)	2.8	2.9	2.7	2.9	3
Left atrium (1.9–4.0cm)	3.7	4	4.2	4.1	4.1
Right ventricule (1.0–2.6cm)	1.6	2.4	2.3	2.2	2.5
Ventricular septal (0.9–1.1cm)	0.9	1.1	1.2†	1.2	0.9
Pulmonary artery systolic pressure (<35mmHg)			26		32
Liver iron concentration (<2.2mg/g dry weight)	3.9	1.2	1.4	2.0	1.4
MRI T2* liver (>12.5ms)	6.7	23.5	20	13.7	19.5
MRI T2* heart (>20ms)	6.5	13.8	45	33.1	43.2
MRI T2* pancreas (>21ms)	9.1	14	17	NA	NA

† Standard flow type pseudo normal left ventricular filling (diastolic dysfunction moderate).

NA: a myolipoma was detected in the left adrenal which impaired the pancreatic measurement in T2* MRI; RM T2*: ressônancia magnética em T2*.

Since then, MRI T2* and echocardiography have shown a complete resolution and recover of cardiac function, as shown in table 2. The patient remains in chelation in order to prevent new deposits of iron using only deferiprone.

## DISCUSSION

Although the mechanism responsible for the accumulation of iron in patients with xerocytosis is still unclear, it is known that patients have considerable deposits of iron in the heart, liver and pancreas, leading to the same clinical manifestations as those found in patients with thalassemia and sickle cell anemia with secondary hemochromatosis^(3,4)^. However, the lack of correlation between levels of ferritin and iron accumulation can be even more deleterious, as the levels found are often low, delaying diagnosis and remedial measures, specifically chelation^(8)^.

MRI has gained acceptance as a model for quantification of total body iron. It has a good cost-benefit ratio, is rapidly performed, reproducible and noninvasive. It can be used in several countries, since technical standards have been developed enough to make the test reliable in the daily clinical management of various disorders that lead to the accumulation of iron. MRI is often used to estimate iron overload in the liver and heart^(7–9)^.

Serum levels of ferritin do not correlate with cardiac deposits and, in the heart, intense tissue toxicity can be observed even without large deposits of iron^(8)^. Cardiac iron cannot be measured by ferritin levels or hepatic iron accumulation, and conventional methods of cardiac evaluation do not detect changes until dysfunctions have already set in^(7,10)^. The use of MRI T2* for the measurement of cardiac iron allows for early identification of patients who require iron chelation, even before the onset of symptoms or echocardiographic findings consistent with systolic dysfunction, allowing for a reduction in mortality related to heart failure^(10,11)^.

MRI T2* is considered the standard procedure for the diagnosis of iron overload^(7)^. It is important to diagnose and treat other hemolytic anemias that lead to iron overload, including xerocytosis, since these patients often present with symptoms of heart failure and diabetes, secondary to hemochromatosis, even without an elevated number of transfusions, or with elevated levels of ferritin still below 500ng/mL. We suggest the regular use of MRI T2* for the diagnosis, monitoring, control of the response to overload, and iron chelation in patients with xerocytosis, and we believe it can be used also in the follow-up of other hemolytic anemia requiring transfusions, even if sporadically. This would allow early diagnosis of iron overload and chelation therapy.
